# Supplementary material for: Abnormal serum levels of liver enzyme markers and related risk factors in type 2 diabetes mellitus patients attending the Buea Regional Hospital, Cameroon
Source: PLoS One. 2025 Jul 21;20(7):e0328974. doi: 10.1371/journal.pone.0328974 (PMC12279140; doi:10.1371/journal.pone.0328974)
Supplement: S1 File — (DOCX) [file pone.0328974.s001.docx]

**QUESTIONNAIRE**

**Assessment of liver function markers in Diabetes patients**

**Instructions:**

***Dear Participants, please tick the appropriate answer and write where needed***

Code: _________ Date: _______________

***Section A: Socio-demographic characteristics***

1. Age: __________
2. Age range:  [21-40[  [40-60[  > 60
3. Gender:  Male;  Female
4. Marital status:  Single  Married  Divorced  Widow
5. Occupation:  Civil servant;  Employee;  Retired
6. Highest Level of Education (Last grade of school completed):  Primary  Secondary  Tertiary (University)

***Section B: Disease history and monitoring***

1. How long have you had diabetes or the year you were diagnosed?  < 5years;  > 5years
2. Have you ever been instructed on diabetes care?  No  Yes
3. Do you have any physical limitations that may affect your ability to perform your self-care?

- Hearing problems  Problems with the use of your hands
- Vision loss (not corrected by glasses or contacts)  Problems with the use of your feet

1. Have you ever been diagnosed, ever been told, or have you had problems with the following?

- High Blood pressure  Kidney disease
- Diabetes retinopathy  Diabetes neuropathy  Cardiovascular diseases

Other health problems: ____________________________________________________

1. Do you regularly check your blood sugar level?  Yes  No
2. If yes, how often do you test?  Once a day  2 or more times a day  Once/Twice a week
3. Are you on diabetes treatment?  No  Yes
4. What is the name of the medication____________
5. Any side effects from the medications that you know of?  No  If yes, what are they?_____________________
6. Do you take any additional nutritional supplements?  Vitamins  Herbal supplements

Others___________________________________________________________________

1. Have you ever forgotten to take your diabetes medication?  No  Yes/How often? _________
2. How important is it to you to take your medicines, where 1 is not important at all and 10 is very important?

1 2 3 4 5 6 7 8 9 10

***Section C: Eating habit and practice of physical activity***

1. Do you regularly drink alcohol? No Yes
2. Are you a tobacco user?  No Yes
3. Have you ever smoked in the past?  No Yes
4. Do you have a current meal plan?  No Yes
5. If so, what is it? __________________________
6. Did you take snack between meals?  No Yes
7. How many liters of water do you drink per day:  1-2L  3-4L 5L and more
8.
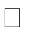
How will you describe your diet?  More of Sugar  More of Vegetables  More of Meat (Proteins)  More of Fats/Oils
9. Do you exercise regularly?  No  Yes
10. If yes, how often do you exercise per week?  < 3 times per week  > 3 times per week

***Thank you for your participation***

**Please do not fill the box bellow (For laboratory and technical use only)**

| **Weight =** | **Height =** | **BMI =** | **Remark:**  Underweight  Healthy weight  Overweight  Obese | | **Blood pressure**  Normal  High | |
| --- | --- | --- | --- | --- | --- | --- |
|  | **Serum enzyme testing** | | | | |  |
|  | **Normal range of values** | **Value obtained (U/L)** | **Remark:**  Normal  High | | | |
| **ALT** |  |  |  |  | |  |
| **AST** |  |  |  |  | |  |
| **ALP** |  |  |  |  | |  |
| **GGT** |  |  |  |  | |  |
